# Changes in Lung Function and Patient-Reported Outcomes in Patients with Idiopathic Pulmonary Fibrosis

**DOI:** 10.1007/s00408-025-00845-z

**Published:** 2025-08-27

**Authors:** Jamie L. Todd, Megan L. Neely, Anne S. Hellkamp, Daniel A. Culver, Justin M. Oldham, Peide Li, Divya C. Patel, Scott M. Palmer

**Affiliations:** 1 Duke Clinical Research Institute, Durham, NC USA; 2 Duke University Medical Center, Durham, NC USA; 3 Department of Pulmonary and Critical Care Medicine, Cleveland Clinic, Cleveland, OH USA; 4 Division of Pulmonary and Critical Care Medicine, University of Michigan, Ann Arbor, MI USA; 5 Boehringer Ingelheim Pharmaceuticals, Inc, Ridgefield, CT USA

**Keywords:** Interstitial lung diseases, Observational study, Pulmonary function tests, Quality of life

## Abstract

We evaluated relationships between changes in lung function and changes in patient-reported outcomes (PROs) in 736 patients with idiopathic pulmonary fibrosis (IPF) enrolled in the IPF-PRO Registry. Weak correlations were observed between changes in percent predicted values for forced vital capacity or diffusing capacity of the lungs (DLco) and changes in St George’s Respiratory Questionnaire (SGRQ) total and activity scores and the 12-item Short Form Survey (SF-12) physical component summary score over 12-month periods. Patients who had a deterioration in SGRQ activity score or SF-12 PCS score of ≥ 5 units had numerically larger declines in lung function than other patients, but the differences were small. The weak relationships observed between changes in lung function and changes in PROs underscore the importance of evaluating both changes in lung function and changes in HRQL in clinical practice and clinical trials.

**Supplementary Information:**

The online version contains supplementary material available at 10.1007/s00408-025-00845-z.

## Introduction

Idiopathic pulmonary fibrosis (IPF) is a progressive fibrosing interstitial lung disease (ILD) characterized by decline in lung function and high mortality [1]. As IPF progresses, patients experience a deterioration in health-related quality of life (HRQL) due to worsening of symptoms and loss of physical function [2–4]. Decline in forced vital capacity (FVC) or diffusing capacity of the lungs for carbon monoxide (DLco) is clearly associated with progression of IPF and mortality [5, 6] and measurement of these parameters is used clinically to assess disease status [7]. However, the relationships between changes in lung function and changes in HRQL in patients with IPF remain uncertain. We assessed the relationships between changes in lung function and changes in HRQL among patients in the IPF-PRO Registry.

## Methods

Patients with IPF that was diagnosed or confirmed at the enrolling center in the previous 6 months were enrolled into the IPF-PRO Registry at 46 sites (Online Resource 1) between June 2014 and October 2018 [8]. Patients were followed prospectively, with data collected as part of routine clinical care, until death, lung transplant, or withdrawal from the registry. Data for this analysis were extracted from the database in March 2023.

HRQL was assessed at enrollment and at follow-up clinic visits using the cough symptoms and cough impact domains of the Cough and Sputum Assessment Questionnaire [9], the EuroQoL 5D and EuroQoL visual analog scale [10], the St George’s Respiratory Questionnaire (SGRQ) [11] and the 12-item Short Form Survey (SF-12) questionnaire [12]. As lung function was measured as part of routine clinical care, measurements of FVC and DLco varied in their frequency and timing. Thus, a joint model based on measurements and visit frequency was used to generate estimates for FVC % predicted and DLco % predicted for each patient for each day during follow-up (Online Resource 2).

First, we assessed the correlation of each patient-reported outcome (PRO) with lung function measures at enrollment (among participants with non-missing values for each pairwise comparison) using the Pearson correlation coefficient (R) (Online Resource 3). The SGRQ activity score showed the best correlations with lung function measures, followed by the SGRQ total score and the SF-12 PCS score. The SGRQ domain (activity, symptoms, impact) scores and total score range from 0 to 100, with higher scores indicating worse HRQL. The SF-12 physical component summary (PCS) score ranges from 0 to 100, with lower scores indicating worse HRQL. SF-12 PCS scores were obtained using the SF-12v2 Standard, US Version 2.0, which scores responses based on 2009 US population norms that have a mean of 50 and a standard deviation of 10. As the SGRQ activity and total scores and SF-12 PCS score showed the best correlations with lung function, we assessed correlations between FVC and DLco % predicted and these PROs at enrollment and after 12 and 24 months of follow-up, and between absolute changes in FVC or DLco % predicted and absolute changes in these PROs from enrollment to 12 months and from 12 to 24 months. Mean changes in FVC or DLco % predicted between enrollment and 12 months and between 12 and 24 months were also calculated across subgroups defined by change in health status over the same interval, *i.e.,* by a ≥ 5-unit change in SGRQ activity score or a ≥ 5-unit change in SF-12 PCS score. A receiver operating characteristic (ROC) curve was used to estimate changes in each lung function measure that best classified the cohort into patients who had versus did not have at least a ≥ 5-unit increase in SGRQ activity score or ≥ 5-unit decrease in SF-12 PCS score. A threshold of 5 units was selected for the SGRQ activity score and SF-12 PCS score as this has been estimated as the minimal clinically important difference for these scores in patients with IPF [13, 14]. The analysis cohort included all patients with ≥ 1 value for the SGRQ activity score between 3 and 27 months after enrollment in the registry.

## Results

Of 1002 patients enrolled in the registry, 736 patients were included in the analysis cohort. At enrollment, median (Q1, Q3) age was 70 (65, 75) years; 539 patients (73.2%) were male; 494 (67.1%) were current or former smokers; 354 (48.1%) took nintedanib or pirfenidone; 96 (13.0%) used oxygen at rest and with activity. Median (Q1, Q3) FVC % predicted was 73.9 (64.0, 85.5) and median (Q1, Q3) DLco % predicted was 44.2 (35.3, 53.2). Median (Q1, Q3) values were 36.7 (23.9, 49.9) and 53.6 (35.8, 71.0) for the SGRQ total and activity scores, respectively, and 40.0 (33.0, 46.7) for the SF-12 PCS score.

**Fig. 1:**
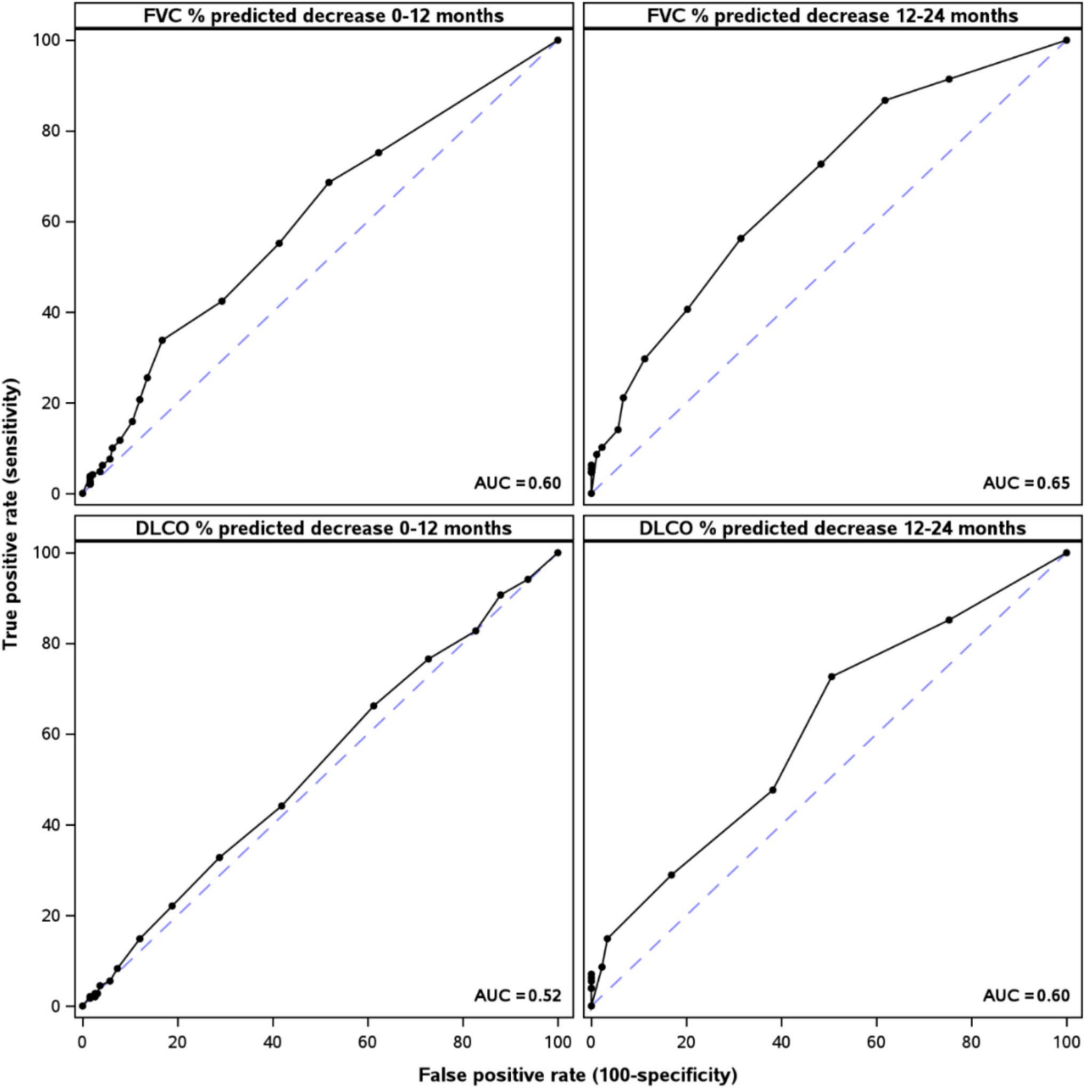
Receiver operating characteristic curves for change in FVC % predicted or DLco % predicted and deterioration in SGRQ activity score (≥ 5-unit increase) and SF-12 PCS score (≥ 5-unit decrease) during the same period. The area under the curve (AUC) indicates the ability of the predictor to discriminate between the outcomes. An AUC of 1 indicates perfect discrimination and an AUC of 0.5 indicates that the predictor is no better than a random guess. A random guess would yield a point along the blue dashed line

Correlations between lung function measures and the selected PROs at each time point were weak to modest (Table 1). Changes in lung function measures and in the PROs between enrollment and 12 months and between 12 and 24 months were weakly correlated (Table 1). Mean changes in lung function measures from enrolment to 12 months and from 12 to 24 months by category of change in a PRO during the same interval are shown in Table 2. Patients who had deterioration in the SGRQ activity score or SF-12 PCS score of ≥ 5 units had numerically larger declines in lung function than those who had improvement of ≥ 5 units or no change, but the differences were small. The best area under the curve achieved in the ROC analyses was 0.65 (Fig. 1), suggesting that no threshold of lung function decline was a good predictor of deterioration in the PRO anchors.

**Table 1:** Correlations between lung function measures and selected PROs

	SGRQ activity score		SGRQ total score		SF-12 PCS score	
	R	R^2^	R	R^2^	R	R^2^
*FVC % predicted*						
Enrollment	− 0.31	0.10	− 0.29	0.08	0.26	0.07
12 months	− 0.37	0.14	− 0.38	0.14	0.28	0.08
24 months	− 0.40	0.16	− 0.37	0.14	0.30	0.09
Change from enrollment to 12 months	− 0.28	0.08	0.08	0.01	0.20	0.04
Change from 12 to 24 months	− 0.25	0.06	− 0.35	0.12	0.35	0.12
*DLco % predicted*						
Enrollment	− 0.32	0.10	− 0.29	0.08	0.24	0.06
12 months	− 0.42	0.18	− 0.39	0.15	0.29	0.08
24 months	− 0.43	0.18	− 0.38	0.14	0.33	0.11
Change from enrollment to 12 months	− 0.15	0.02	0.11	0.01	0.06	0.00
Change from 12 to 24 months	− 0.20	0.04	− 0.24	0.06	0.23	0.05

**Table 2:** Mean change in lung function measures by category of change in PRO during the same time interval

	Enrollment to 12 months	12 to 24 months
Mean change in FVC % predicted (n)		
SGRQ activity score		
Improvement (decrease ≥ 5)	− 1.2 (143)	− 2.0 (55)
No change (|change|< 5)	− 1.3 (91)	− 2.3 (50)
Deterioration (increase ≥ 5)	− 3.1 (237)	− 3.6 (103)
SF-12 PCS score		
Improvement (increase ≥ 5)	− 0.4 (81)	− 1.2 (27)
No change (|change|< 5)	− 2.0 (227)	− 2.6 (118)
Deterioration (decrease ≥ 5)	− 3.3 (154)	− 4.3 (59)
Mean change in DLco % predicted (n)		
SGRQ activity score		
Improvement (decrease ≥ 5)	− 4.5 (143)	− 1.2 (55)
No change (|change|< 5)	− 4.6 (91)	− 1.3 (50)
Deterioration (increase ≥ 5)	− 4.9 (237)	− 2.0 (103)
SF-12 PCS score		
Improvement (increase ≥ 5)	− 4.6 (81)	− 1.3 (27)
No change (|change|< 5)	− 4.7 (227)	− 1.3 (118)
Deterioration (decrease ≥ 5)	− 4.8 (154)	− 2.2 (59)

## Discussion

We used data from the prospective multicenter IPF-PRO Registry to evaluate relationships between lung function measures and PROs in a contemporary cohort of patients with IPF. We found that there was only a weak relationship between decline in lung function and deterioration in HRQL assessed using PROs. No threshold of lung function decline performed well at classifying patients into those with versus without a concurrent deterioration in HRQL.

Weak correlations between lung function measures and PROs, and even more so between changes in lung function measures and changes in PROs, have also been observed in studies assessing the psychometric properties of PROs in patients with IPF [15, 16]. Consistent with this, clinical trials of therapies for IPF have demonstrated a significant reduction in the rate of FVC decline, but no significant difference in changes in PROs [17, 18]. These data suggest that physiologic measures and PROs assess different aspects of the impact of IPF. The impact of a given severity of disease on a patient’s HRQL may be influenced by a patient’s personal perspectives and lifestyle: a recent study in 50 patients with IPF found poor agreement between physicians’ ratings of patients’ clinical status, which were closely related to DLco % predicted, and the patients’ ratings, which were likely based on a more complex assessment of their overall well-being [19]. Further, as baseline lung function may affect the ability of a patient to perceive a decline in FVC, the same absolute change in FVC may have a different impact on symptoms or functional capacity in patients with different severities of disease. These observations support the evaluation of both changes in lung function and changes in HRQL in clinical practice and clinical trials, as recommended in a recent symposium on meaningful endpoints for use in clinical trials in IPF [20].

Understanding the minimal important change to the patient (MICP) of clinical measures, *i.e.,* the smallest difference perceived as important by patients, would assist clinicians in the interpretation of changes. However, a prerequisite to performing such an analysis would be at least a moderate correlation between changes in the anchor and changes in the indicator of interest [21, 22]. In our study, the rather weak correlations between changes in lung function measures and changes in PROs meant that anchors based on these PROs would be inadequate for determining MICPs of lung function measures. It is possible that PROs developed in patients with ILDs, such as the Living with Pulmonary Fibrosis questionnaire [23, 24], may be better correlated with lung function, but this requires further investigation.

In conclusion, these analyses of data from the IPF-PRO Registry suggest that in patients with IPF, there is only a weak relationship between changes in lung function and changes in PROs. Our findings underscore the importance of including PROs in clinical trials, given that lung function changes may not correspond to patients’ experience of their disease.

## Supplementary Information

Below is the link to the electronic supplementary material.

Supplementary file1 (DOCX 475 kb)

## Data Availability

All the data relevant to the study are included in the article.
