# Supplementary material for: Changes in Lung Function and Patient-Reported Outcomes in Patients with Idiopathic Pulmonary Fibrosis
Source: Lung. 2025 Aug 27;203(1):90. doi: 10.1007/s00408-025-00845-z (PMC12390864; doi:10.1007/s00408-025-00845-z)
Supplement: Supplementary file 1 — Supplementary file1 (DOCX 475 kb) [file 408_2025_845_MOESM1_ESM.docx]

**Supplementary information**

**Online Resource 1: Principal investigators and enrolling centers**

Albert Baker, Lynchburg Pulmonary Associates, Lynchburg, VA; Scott Beegle, Albany Medical Center, Albany, NY; John A Belperio, University of California Los Angeles, Los Angeles, CA; Rany Condos, NYU Medical Center, New York, NY; Francis Cordova, Temple University, Philadelphia, PA; Brian Southern (formerly Daniel A Culver), Cleveland Clinic, Cleveland, OH; Daniel Dilling, Loyola University Health System, Maywood, IL; John Fitzgerald (formerly Leann Silhan), UT Southwestern Medical Center, Dallas, TX; Kevin R Flaherty, University of Michigan, Ann Arbor, MI; Kevin Gibson, University of Pittsburgh, Pittsburgh, PA; Mridu Gulati, Yale School of Medicine, New Haven, CT; Kalpalatha Guntupalli, Baylor College of Medicine, Houston, TX; Nishant Gupta, University of Cincinnati Medical Center, Cincinnati, OH; Amy Hajari Case, Piedmont Healthcare, Atlanta, GA; David Hotchkin, The Oregon Clinic, Portland, OR; Tristan J Huie, National Jewish Health, Denver, CO; Robert J Kaner, Weill Cornell Medical College, New York, NY; Hyun J Kim, University of Minnesota, Minneapolis, MN; Lisa H Lancaster (formerly Mark Steele), Vanderbilt University Medical Center, Nashville, TN; Joseph A Lasky, Tulane University, New Orleans, LA; Doug Lee, Wilmington Health and PMG Research, Wilmington, NC; Timothy Liesching, Lahey Clinic, Burlington, MA; Randolph Lipchik, Froedtert & The Medical College of Wisconsin Community Physicians, Milwaukee, WI; Jason Lobo, UNC Chapel Hill, Chapel Hill, NC; Tracy R Luckhardt (formerly Joao A de Andrade), University of Alabama at Birmingham, Birmingham, AL; Yolanda Mageto (formerly Howard Huang), Baylor University Medical Center at Dallas, Dallas, TX; Marta Kokoszynska (formerly Yolanda Mageto, Prema Menon), Vermont Lung Center, Colchester, VT; Lake Morrison, Duke University Medical Center, Durham, NC; Andrew Namen, Wake Forest University, Winston Salem, NC; Justin M Oldham, University of California, Davis, Sacramento, CA; Tessy Paul, University of Virginia, Charlottesville, VA; David Zhang (formerly Anna Podolanczuk, David Lederer, Nina M Patel), Columbia University Medical Center/New York Presbyterian Hospital, New York, NY; Mary Porteous (formerly Maryl Kreider), University of Pennsylvania, Philadelphia, PA; Rishi Raj (formerly Paul Mohabir), Stanford University, Stanford, CA; Murali Ramaswamy, PulmonIx LLC, Greensboro, NC; Tonya Russell, Washington University, St. Louis, MO; Paul Sachs, Pulmonary Associates of Stamford, Stamford, CT; Zeenat Safdar, Houston Methodist Lung Center, Houston, TX; Shirin Shafazand (formerly Marilyn Glassberg), University of Miami, Miami, FL; Ather Siddiqi (formerly Wael Asi), Renovatio Clinical, The Woodlands, TX; Reginald Fowler (formerly Barry Sigal), Salem Chest and Southeastern Clinical Research Center, Winston Salem, NC; Mary E Strek (formerly Imre Noth), University of Chicago, Chicago, IL; Hiram Rivas-Perez (formerly Jesse Roman, Sally Suliman), University of Louisville, Louisville, KY; Jeremy Tabak, South Miami Hospital, South Miami, FL; Rajat Walia, St. Joseph’s Hospital, Phoenix, AZ; Timothy PM Whelan, Medical University of South Carolina, Charleston, SC.

**Online Resource 2: Statistical methods for estimation of lung function measures**

In the IPF-PRO Registry, pulmonary function test (PFT) values were collected by abstracting data from the medical record; there were no mandated study visits. The time points at which PFT values exist in the registry database vary widely from one patient to another and there can be long gaps between measurements. The frequency of PFT measurement may be related to patients’ health status, (*e.g.,* sicker patients may be more likely to be tested) and the trajectories of PFTs in patients who leave the registry due to worsening health may be different from those of patients who leave for other reasons. Thus, to generate unbiased estimates of lung function measures, joint models simultaneously consider visit patterns, PFT measurements, and terminal events (worsening health). For developing the joint models, the analysis cohort included all patients who had ≥1 PFT measurement (forced vital capacity [FVC] or diffusing capacity of the lungs for carbon monoxide [DLco]) between 30 days before enrollment through to the end of follow-up. Models were used to generate predicted values for all patients.

To account for informative censoring in modeling, we used the outcome of terminal event, defined as death, lung transplant, entry into hospice care, or withdrawal from the registry due to worsening IPF. Patients who did not have a terminal event were censored at the time of last follow-up, which was generally their last interaction with a healthcare system. For the joint models, we used a 48-month follow-up period for PFTs, a length that balanced having a reasonable density of PFT measurements, to allow for the most informative longitudinal modeling, with the censoring that occurs with terminal events. For estimation of lung function measures, we used the same follow-up period for each patient, *i.e.,* from enrollment until terminal event or 48 months (whichever was first).

We used the joint modelling approach described by Liu et al.^1^ This joint model included three sub-models (one for measurement frequency, one for lung function values, one for terminal events) linked by common random effects. The PFT sub-models did not include covariates, but the measurement frequency and terminal events models did. These were (all assessed at enrollment): age, race/ethnicity, body mass index, FVC % predicted, DLco % predicted, and supplemental oxygen use. Only race/ethnicity had missing values; these were imputed using multiple imputation with fully conditional specification. In the PFT sub-models, we used restricted cubic spline fits for time, and subject-level random effects were attached to intercept and both linear and nonlinear elements of the time terms. This allows the shape of each subject’s trajectory to be flexible with respect to time. The models included three random effects: intercept, linear time term, and a single random effect for both nonlinear terms; and the covariance between the linear and non-linear random effects. Initial evaluation indicated no evidence of a shared random effect between the PFT and terminal event sub-models. Thus, the joint model that included subject-level random effects was a two-part model consisting of PFT and visit frequency sub-models. The final PFT predictions were generated from this model.

**Reference**

1. Liu L, Huang X, O’Quigley J (2008) Analysis of longitudinal data in the presence of informative observational times and a dependent terminal event, with application to medical cost data. Biometrics 64(3):950–958. <https://doi.org/10.1111/j.1541-0420.2007.00954.x>

**Online Resource 3: Correlations between PROs and lung function measures at enrollment in participants with non-missing values for the pairwise comparison.**

**
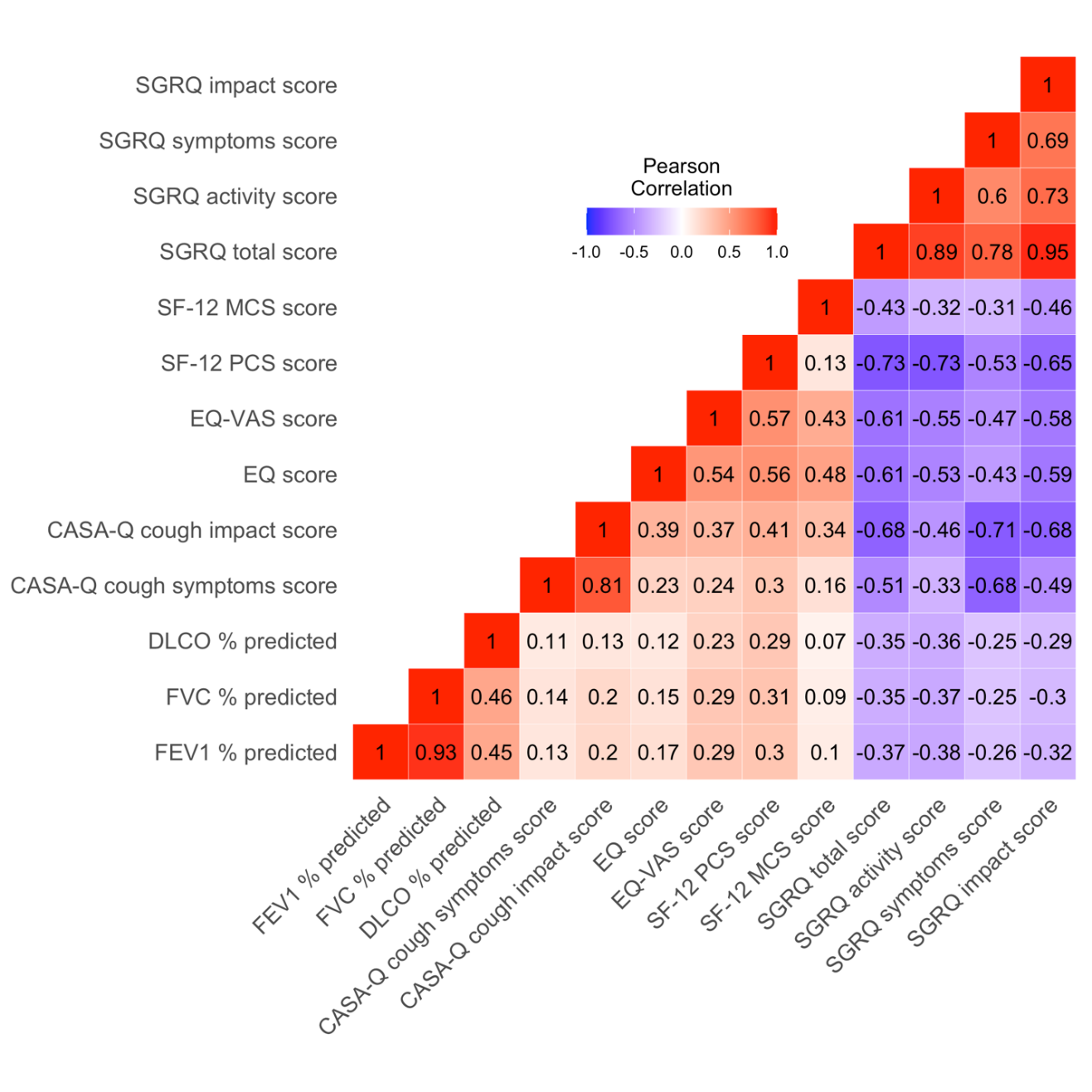
**
